# Emotional Induction Through Music: Measuring Cardiac and Electrodermal Responses of Emotional States and Their Persistence

**DOI:** 10.3389/fpsyg.2019.00451

**Published:** 2019-03-06

**Authors:** Fabiana Silva Ribeiro, Flávia Heloísa Santos, Pedro Barbas Albuquerque, Patrícia Oliveira-Silva

**Affiliations:** ^1^School of Psychology (CIPsi), University of Minho, Braga, Portugal; ^2^Faculty of Education and Psychology (CEDH/HNL), Universidade Católica, Porto, Portugal; ^3^School of Psychology, University College Dublin, Dublin, Ireland

**Keywords:** emotional induction, music, emotion, skin conductance level, heart rate, emotional persistence

## Abstract

Emotional inductions through music (EIM) procedures have proved to evoke genuine emotions according to neuroimaging studies. However, the persistence of the emotional states after being exposed to musical excerpts remains mostly unexplored. This study aimed to investigate the curve of emotional state generated by an EIM paradigm over a 6-min recovery phase, monitored with valence and arousal self-report measures, and physiological parameters. Stimuli consisted of a neutral and two valenced musical excerpts previously reported to generate such states. The neutral excerpt was composed in a minimalist form characterized by simple sonorities, rhythms, and patterns; the positive excerpt had fast tempo and major tones, and the negative one was slower in tempo and had minor tone. Results of 24 participants revealed that positive and negative EIM effectively induced self-reported happy and sad emotions and elicited higher skin conductance levels (SCL). Although self-reported adjectives describing evoked-emotions states changed to neutral after 2 min in the recovery phase, the SCL data suggest longer lasting arousal for both positive and negative emotional states. The implications of these outcomes for musical research are discussed.

## Introduction

Emotional responses have been reported as one of the primary motivations to listen to music (Schäfer et al., 2013; Shifriss et al., 2015; Reybrouck and Eerola, 2017), and several studies have shown that music can evoke genuine basic emotions, such as happiness, sadness, and fear (Västfjäll, 2002; Fritz et al., 2009; Egermann et al., 2015).

In this context, emotion is a set of homeostasis-related body alterations that involve changes in the brain activations (Damasio, 2004). Therefore, an effective emotional stimulus can trigger hormonal and autonomic responses that prepare the body for various actions or complex behaviours. The most common model of emotion used across studies is the valence–arousal since it enables a dimensional description (Eerola and Vuoskoski, 2011; van der Zwaag et al., 2011). The valence designates the extent to which an emotional stimulus is positive/happy or negative/sad. This dimension is usually assessed by self-report measures, while arousal refers to the degree of excitation elicited by a stimulus in an individual, which produces a physiological measurable increase or decrease of the autonomic system activation. The arousal dimension is often measured also by self-reported measures and physiological parameters, for instance, skin conductance level (SCL) and heart rate (HR) response (Lang et al., 1997; Russell, 2003; Mauss and Robinson, 2009).

Specifically, the SCL is referred to as a reliable measure of the sympathetic nervous system (Boucsein et al., 2012). In fact, the SCL increment is associated with higher physiological arousal, which is usually caused by emotional stimuli (Lang et al., 1993; Kreibig et al., 2007). Furthermore, HR decelerations are associated with unpleasant stimuli (Palomba et al., 1997) and increased attentional responses. For instance, if a negative arousing stimulus is more attention demanding, it may elicit a deceleration of HR (Bradley and Lang, 2000; Sammler et al., 2007; Taelman et al., 2011).

Some studies have already benefited from physiological measures confirming the effectiveness of music in eliciting genuine emotions (Krumhansl, 1997; Khalfa et al., 2002; Etzel et al., 2006; Lundqvist et al., 2009; White and Rickard, 2016; Mori and Iwanaga, 2017). Krumhansl (1997) used a range of physiological measures, including HR and SCL, while participants were listening to 3-min songs, intended to induce sad, fearful, and happy emotions. The author found that Albinoni’s Adagio in G Minor and Barber’s Adagio pour Cordes songs, rated as sad excerpts by participants on a rating from 0 to 8 on valenced scales, produced slower HR, and decreased SCL in comparison to happy excerpts, namely La Primavera (Spring) from The Four Seasons (composed by Antonio Vivaldi) and Midsommarvaka (composed by Hugo Alfven). Congruently, Etzel et al. (2006) when using erudite music found HR deceleration during a sad excerpt with length varying from 74 to 189 s and HR acceleration during fear inductions.

Khalfa et al. (2008) investigated whether psychophysiological differences between happy and sad music would be attributable to tempo or rhythm variations. At a psychophysiological level, the authors showed that 1-min happy excerpts with the explicit instruction to participants focus on feeling the emotions evoked by the musical stimuli, were able to increase the *change values* signals (calculation of mean SCL activity during musical listening minus the mean signal of recovery phase) of SCL activity compared to the sad ones. However, no HR differences between sad and happy music were observed. According to the authors, more extended excerpts are more likely to induce HR differences in response to happy and sad music. This argument is explained by the musical expectations, which are based on the listener’s prior experiences with music. Like language, music comprises perceptually discrete elements that are preorganized according to rules and emotional reaction happen when expectations are somehow disrupted in the excerpts compositions (Juslin and Västfjäll, 2008).

Consistently, Lundqvist et al. (2009) investigated the impact of happy and sad pop songs on a variety of physiological measures, including HR, and SCL. In this study, they noticed that the happy song elicited significantly greater SCLs than the sad one. Regarding the HR, a deceleration–acceleration activity pattern was observed at the beginning of the emotional stimuli, regardless of its valence. The authors explained these HR findings as an orienting response that might be associated with a shift on the participant’s attention toward the task.

When it comes to the musical features that may convey emotions to listeners, some authors have advocated that the autonomic responses underlying the emotional response induced by musical stimuli could result from intrinsic and/or extrinsic musical properties (Sloboda and Juslin, 2001). The intrinsic properties of music are those that elicit an emotional response in the listener as a direct consequence of musical structural features, such as intensity, tempo, and mode. The extrinsic properties are the emotional responses elicited as a consequence of the association of the musical structure with previous personal events, experiences, or contextual associations of the sound, i.e., how subjects relate music to a particular setting, context, or circumstance (Sloboda and Juslin, 2001). Focusing on the intrinsic properties, music evaluated as happy is typically composed in major mode and fast tempo, while sad music is slower and written in the minor tone (Hodges, 2010; White and Rickard, 2016; Taruffi et al., 2017). Congruently, some studies found positive correlations between valence–arousal parameters and musical structures (Balkwill and Thompson, 1999; Egermann et al., 2013).

Nevertheless, fast *tempo* is often related to highly arousing music, while a piece of slow *tempo* music is linked to low arousal, and these different rhythmic patterns would influence the listener emotional perception (Gabrielson and Juslin, 2003). Physiological functioning tends to mimic the musical expression, i.e., the rhythmic patterns, and through afferent physiological feedback, it elicits the emotion conveyed by the *tempo* (Scherer and Zentner, 2001; Dibben, 2004). Then, specifically for music, the positive (happy) and negative (sad) emotions effects in arousal might depend on musical elements composing the excerpt and not only to the quality of the emotion.

Current studies also have found that sad songs can evoke tears and chills; in which tears were accompanied by HR acceleration, while chills showed to increase electrodermal activity and subjective arousal (Mori and Iwanaga, 2017). Additionally, women were reported as being more likely to feel chills with increased SCL than men (Panksepp, 1995). Nevertheless, sad songs can also be reported as unpleasant and not relaxing, which can produce increases in the SCL (Baumgartner et al., 2006; Nater et al., 2006). Moreover, the ability of sad music to generate pleasure or displeasure might be influenced by its esthetics and also by personality, mood, and learned associations of who listen to it (Sachs et al., 2015).

Assuming that music can generate happy and sad emotions, it is possible to argue that valenced music could also generate moods. In this perspective, the mood can be defined as a general affective background that persists over time without a specific stimulus (Beedie et al., 2005). To assess the musical influence on mood, Eich and Metcalfe (1989) produced continuous musical induction throughout a 90-min session, while participants performed an encoding and retrieval task. Mood states were measured throughout different moments in the session. Results showed that, in the beginning, positive (or negative) songs made participants feel happy (or sad) as measured by self-report, and with repeated exposure, the mood manipulation lost some of its intensity. However, continuous music induction produced statistically reliable data since there were still differences between the positively and negatively induced participants at the end of the experiment, which show relatively stable changes by musical mood induction.

The literature on the persistence of the emotional effects after musical exposure on HR and SCL signals are scarce, yet critical. If an induced emotion persists after its induction, one may also expect to observe a variation in physiological responses of the subjects induced by music. One of the few studies investigating the persistence of emotions used four different valence–arousal film clips with its original soundtracks to induce positive high-arousal, positive low-arousal, negative high-arousal, and negative low-arousal. These mood inductions lasted 10 min; they were followed by a computer task (online shopping) aiming to observe whether induced emotions would last throughout the cognitive task. The authors reported lower SCL for the negative videos during the accomplishment of the task. In contrast, the HR results for those participants who watched the two positive videos did not present significant differences. Also, after approximately 9 min of the computer task, no self-reported arousal effects were observed (Gomez et al., 2009).

Another study carried out by Kuijsters et al. (2016), similarly selected sad videos with their original soundtracks as inductors for 10 min and subsequently assessed mood maintenance throughout 8 min at recovery phase, measuring emotional states through self-report, HR, and SCL. The results showed that SCL was above the baseline until the fourth minute of the recovery phase, and the HR results were similar to the baseline. The authors observed that the self-reported arousal decayed fast after the first minute and slowly returned to baseline. Conversely, the SCL suggested longer-lasting arousal effects, as such the above-quoted study (Gomez et al., 2009).

Although the previous two studies investigated the effects of emotional persistence, they included videos with soundtracks to induce emotion. As demonstrated by other authors, this junction of video and audio stimuli may lead to mixed emotional messages and elicitation, such as simultaneous positive and negative emotions, since it requires an added degree of elaborative processing when compared to non-combined stimuli (Koelstra et al., 2010).

According to Koelsch (2010), a particular advantage of using music to evoke emotions, in relation to other stimuli, is that it enables researchers to study positive emotions (e.g., fun and enjoyment) or negative emotions (e.g., sadness), which are challenging to evoke in experimental settings. Moreover, such emotions evoked by musical stimuli are meaningful to music therapy, and rehabilitation since music allows emotions to be experienced and communicated indirectly, i.e., without verbalization.

Despite the remarkable body of work providing evidence for the effectiveness of music in inducing emotions and moods (Krumhansl, 1997; Khalfa et al., 2002; Etzel et al., 2006; Lundqvist et al., 2009; White and Rickard, 2016; Mori and Iwanaga, 2017), little is known about how emotions evoked through music evolve over time. As shown by the cited studies, a significant difference in valence and arousal ratings after musical exposure is used as an indication of successful induction, but scarcely any quantification of its persistence has been detailed in the literature (Koelsch, 2010). For this reason, the present study aimed to investigate the persistence of sad and happy emotions evoked by an Emotional Induction through Music (EIM) procedure originally designed for the current study comprised by erudite instrumental music, and analysis of the progress of the emotions until a return to baseline after the EIM.

We hypothesized that the EIM procedure would be effective in inducing positive and negative emotions, happy and sad, respectively, taking into account self-reported mood responses and physiological measures. Specifically, positive (happy) emotions evoked through music should produce higher arousal, while negative (sad) emotions should cause lower arousal in contrast to neutral states. We expected to detect these differences by the physiological measurement, and consequently observe longer lasting moods during the recovery phase after both EIM procedures.

## Materials and Methods

### Participants

Thirty-eight Portuguese college students participated in this investigation in exchange for partial course credit. They were screened and ruled out if they reported any history of neurological or psychiatric disorders or severe levels of depression or anxiety, or hearing or visual impairments that could not be corrected. Four people out of 38 participants were ruled out: two of them presented high levels of anxiety; the other two were excluded due to procedural problems. The final sample included 34 healthy participants, as displayed in Table 1, with a mean age of 23.38 (*SD* = 4.68; 18 females). None of them had received formal musical training and were naïve to the purpose of the experiment. This study was approved by the University of Minho Ethics Commission in accordance with the Code of Ethics of the World Medical Association (Declaration of Helsinki) for experiments involving humans.

**Table 1:** Mean scores and standard deviations (in parenthesis) for screening.

Screening (*n* = 34)	Mean (*SD*)
Beck Anxiety Inventory	7.32 (4.87)
Beck Depression Inventory	4.68 (3.11)
**Profile of mood states**	
Tension	5.23 (3.42)
Depression	3.18 (3.51)
Anger	3.53 (3.56)
Vigor	13.32 (3.47)
Fatigue	5.29 (4.10)
Confusion	1.47 (2.03)
Total	105.38 (14.52)

### Materials

#### Screening

*Beck Anxiety Inventory* (Beck and Steer, 1993). It is a 21-item self-report scale that measures the intensity of anxiety symptoms. The items must be assessed by the participant on a four-point scale: 0 – nothing (not at all); 1 – mild (mildly, but it didn’t bother me much); 2 – moderate (moderately, it wasn’t pleasant at times), and 3 – severe (severely, it bothered me a lot). The total score is calculated by the sum of each response item and can vary between 0 and 63. Higher total scores indicate more severe anxiety symptoms. The standardized Portuguese cut-offs are: minimal anxiety level: 0–10 points; mild level: 11–19 points; moderate level: 20–30 points; and severe level: 31–63 scores (Oliveira and Yoshida, 2009).

*Beck Depression Inventory* (Beck et al., 1996). The Beck Depression Inventory is a 21-item self-application instrument that measures depression severity symptoms in adults and adolescents at age 13 years or older. Items are measured on a four-point scale in which each answer is scored on a scale value of 0–3 with a maximum score of 63. Higher total scores designate severe depressive symptoms. The standardized Portuguese cut-offs are: minimal depression: 0–13 points; mild depression: 14–19 points; moderate symptoms: 20–28 points; and severe depression: 29–63 points (Gomes-Oliveira et al., 2012).

*Profile of Mood States* (McNair et al., 1992). It contains 42 adjectives that measure six mood dimensions of adults aged 18 years and older: tension, depression, anger, vigor, fatigue, and confusion. The adjectives are assessed on a scale value of 0–5 points. The Tension (T) dimension is composed of adjectives describing increases of musculoskeletal tension and preoccupations; Depression (D) represents an emotional state of discouragement, sadness, unhappiness, and loneliness; Anger (A) dimension corresponds to a mood of anger and dislike compared to the others; Vigor (V) is related to a physical energy state and psychological vigor; Fatigue (F) represents a state of tiredness, inertia, and low energy; and finally, Confusion (C) is characterized by a state of confusion and low lucidity. Moreover, a total mood disturbance result is calculated adding the five negative dimensions (T+D+A+F+C), subtracting the Vigor scale result, and summing a constant of 100 to avoid a negative result. Normative values for the Portuguese population were published by Viana et al. (2001).

#### Emotional Induction Through Music Procedure (EIM)

The emotional induction procedure was carried out with erudite music selected from previously published works (Västfjäll, 2002; Grewe et al., 2010). However, these studies did not specify the arousal levels of songs. Besides, no valence–arousal standardized ratings of erudite music were published for the Portuguese population. For this reason, we carried a pilot study including three positive, four negative, and seven neutral excerpts that allowed us to identify which musical excerpt was the most effective in each condition (provided in the Supplementary Material).

##### Musical excerpts

The length of each music excerpt was precisely 3 min, and each musical excerpt was edited using Audacity^®^ 2.1.2 to avoid startling participants. “Fade out” and “fade in” were applied when necessary and normalized to the same Root Mean Square loudness level. The musical excerpt used for negative induction was *Albinoni “Adagio” composed in G-minor with 3/4 time signature*. For positive mood induction, we used *Bach “Brandenburg concert n∘2” composed in F-Major with 2/2 time signature.* The neutral excerpt was *“Steve Reich-Variations for winds, strings, and keyboard” composed in C-minor/C-flat, and B-major, with varied time signatures* (for more details regarding excerpts editions see Supplementary Material).

##### Valence–arousal self-report measure

Participants rated their emotional state on the valence dimension selecting one out of nine adjectives (adapted from Plutchik, 2001). There were three adjectives related to positive mood (*happy, excited*, and *euphoric*), three to negative mood (*sad, melancholic*, and *distressed*), each one corresponding to a low, medium, or high-intensity level, respectively. We also selected three adjectives to rate neutral mood (*neutral, indifferent*, and *unresponsive*)^1^. Although the valenced adjectives were rated as low, medium, and high intensities, participants were not informed about these intensities.

The arousal dimension was measured immediately after valence, in which participants were requested to rate the arousal of the previously selected adjective on a 7-point analog scale about how they felt at that moment. One corresponded to “I feel very little aroused” and 7 to “I feel very much aroused.” Moreover, to assess ratings of musical pleasantness, a 7-point scale was also included, with 1 meaning unpleasant and 7 meaning very pleasant. All self-reported measurement were computer-based administered using SuperLab 4.5 Software (Cedrus Corporation, San Pedro, CA, United States).

For this study, induction was achieved whenever participants chose one of the three adjectives congruent with the EIM condition (e.g., “this song made me feel … sad,” and the condition of the EIM was negative/sad music). On the other hand, unachieved induction refers to incongruent responses to the induction condition (e.g., “In general this song made me feel … neutral,” and the condition of induction applied was a positive/happy excerpt).

The mood self-report measure was complemented with physiological measurements, i.e., the cardiac (measured by the heart rate – HR) and electrodermal (measured by the skin conductance level – SCL) responses, which are non-invasive measurements.

#### Psychophysiological Measures

A sampling rate of 1000 Hz was chosen for both HR and SCL channels. The psychophysiological measures were recorded using the wireless BioNomadix System (BN-PPGED module for the SCL and BN-RSPEC module for the HR) (BIOPAC Systems Inc., Santa Barbara, CA, United States). The acquisition system was connected to a computer running AcqKnowledge 4.4 software.

##### Skin conductance level

The disposable silver/silver chloride electrodes (BIOPAC Type EL 507) were attached to the palmar surface of the medial phalanges of the index and middle fingers of the non-dominant hand. Then, the BioNomadix transmitter was placed on the participants’ non-dominant wrist by the experimenter. The transmitter passes a constant voltage of 0.5 V between the two sensors and transfers the difference in charge (i.e., the conductance afforded by the sweat glands on the palm) back to the BioNomadix data acquisition unit. The raw SCL data were filtered using the recommended standard filter settings for the acquisition device, an FIR low-pass Blackman filter of 1 Hz with the number of coefficients set at 4000 (Coutinho et al., 2017). Then, 1-min epoch means (for the 3 min during each MIP and the 6 min during recovery phase) were calculated for each participant and exported to SPSS for further analysis.

##### Heart rate

The HR measured in beats per minute was achieved from participants’ raw Electrocardiogram (ECG) using an adjusted three-electrode Lead-II configuration. The HR electrodes were filled with an electrode gel that is specifically intended for use in the recording of bioelectrical potentials. The disposable Ag–AgCl electrodes (BIOPAC Type EL 503) were placed on the participants’ left acromial and sternal end of the clavicle, and a third one on the left spine of the scapula and a transmitter was attached to a belt placed around the participants’ thorax region. Before electrode placement, the skin was cleaned with alcohol and dried with cotton to diminish impedance and to improve signal quality. The raw ECG data were filtered using the recommended standard filter settings for the acquisition device, an IIR high-pass filter of 1 Hz and an IIR low-pass filter set at 35 Hz (Coutinho et al., 2017). HR was calculated offline from the filtered ECG trace using the Acknowledge 4.4 software. Furthermore, within-subject means were calculated based on 1-min epochs within each period of interest for the HR measure, such as for the SCL. The data were exported to SPSS for further analysis.

### Procedure

The experiment was presented to the participants as a study to explore the physiological correlates of emotion and memory in adults, but they were not informed about the specific aims of the study to avoid biased responding. Besides, participants were told that physiological responses would be recorded during the tasks and received basic information on the psychophysiological devices (BIOPAC equipment). Then, participants were asked to sign a written consent form; fill out a demographic questionnaire; and the anxiety, depression, and mood scales.

The electrodes were placed and recording started subsequently. After a 3-min waiting period (Baseline) participants were asked to choose one of the nine adjectives (balanced per valences), followed by a 1–7 analogic scale to assess arousal (the valence–arousal self-report measure). The initial EIM instruction was then displayed on the computer screen, instructing participants to close their eyes as they listened to the song, to increase emotionality (Lerner et al., 2009). Next, they proceeded to the first of the three EIMs. The musical excerpts were presented via headphones at a comfortable volume level (below 60 dB). A valence–arousal self-report measure followed each EIM and by a 6-min silence (Recovery phase), in which participants were asked to wait in silence for the next task. During the recovery phase, participants were requested to evaluate their emotional state with the valence–arousal self-report measure every 1 min.

**FIGURE 1:**
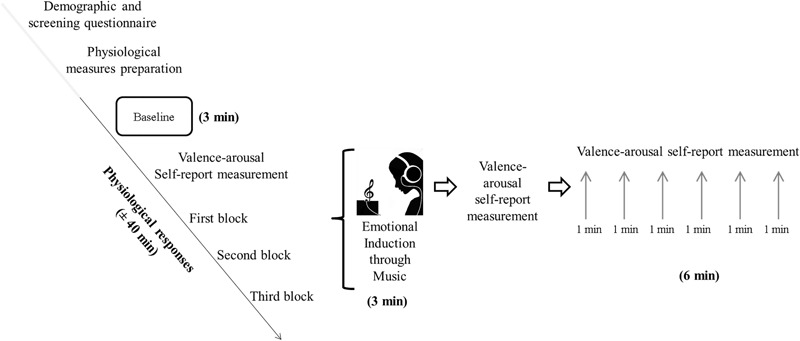
Scheme of the experimental procedure including the first block.

Once the first block (EIM plus recovery phase) was completed, participants were immediately induced for a second and third time (blocks 2 and 3) following the same steps as previously described. The experimental procedure is depicted in Figure 1.

The order of the three musical excerpts of the EIM was counterbalanced between subjects (negative–neutral–positive, neutral–positive–negative, or positive–negative–neutral). The physiological measures were continuously recorded during all moments of the procedure, and the total length of the experiment was around 40 min per participant. All participants were assessed individually.

## Results

We will first report an analysis of the valence–arousal self-reports for all the EIM conditions, including the number of participants congruently induced by the EIM procedure, to observe its effectiveness. Second, we will report exploratory analyses of the valence–arousal self-reports data immediately after each EIM condition and after each minute of recovery phase for those participants induced congruently, and the SCL and HR results during baseline and each EIM condition. Finally, we will report valence–arousal self-reports, SCL, and HR data of those participants induced congruently on the positive and negative conditions of the procedure and the results during the 6-min recovery phase.

### EIM Efficacy

**FIGURE 2:**
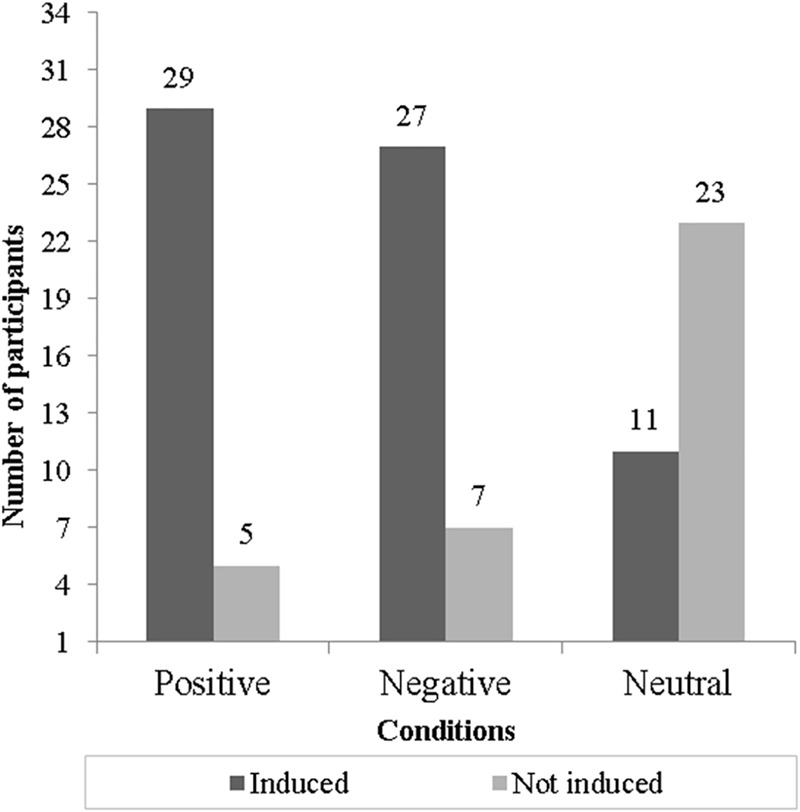
Induction effectiveness for each condition (bars show the number of participants “induced” or “not induced” congruently to each one of three conditions).

According to the valence–arousal self-report measurement, it was possible to detect that positive and negative EIM conditions were effective since they achieved more than 79% of congruent emotional responses. The neutral EIM condition was not effective since only 32% of participants were induced. The effectiveness of each EIM condition is displayed in Figure 2.

Thus, we observed that only four participants were congruently induced across the three conditions, while the other 24 participants were induced congruently by the positive and negative EIM conditions. After the exclusion of non-induced participants, we certified that the conditions were counterbalanced to avoid presentation order effects. We carried out two separated sets of exploratory analyses: the first included the SCL and HR responses for the four participants congruently induced across the positive, negative, and neutral EIM conditions, and the second comprising the SCL and HR responses for those subjects inconsistently induced by the positive and negative EIM in order to observe possible variations in physiological signals.

### Valence–Arousal Self-Report, SCL, and HR Responses for Participants Congruently Induced Across EIM Conditions

Table 2 displays the valence–arousal self-report descriptive results for the four congruently induced participants and shows that the adjectives related to positive and negative EIM conditions change quickly for the neutral ones after the first minute, while the neutral EIM adjectives are constant over time.

**Table 2:** Number of participants that remained induced and mean arousal ratings after EIM and during 6 min recovery phase assessed by the self-report.

	After EIM	1 min	2 min	3 min	4 min	5 min	6 min
**Conditions (number of participants that remained induced)**		
Positive	4	4	2	1	0	0	0
Negative	4	1	0	0	0	0	0
Neutral	4	4	4	4	4	4	4
**Arousal (mean rates of the four participants)**		
Positive	5	3.8	3.5	4	0	0	0
Negative	3.5	3	0	0	0	0	0
Neutral	4	3.8	4.5	3.8	4	4	4.5

Nevertheless, non-parametric tests were used because the data did not assume normal distributions or homogeneity of the variances. The Friedman test rendered no significant results for the HR responses, χ^2^(3) = 0.60, *p* = 0.89. However, we observed a significant difference between conditions for the SCL responses, χ^2^(3) = 10.20, *p* = 0.02. The *post hoc* non-parametric Wilcoxon signed-rank test was used to compare the baseline scores with each EIM condition reported with a Bonferroni correction for multiple tests resulting in a significance level of =0.016 (0.05/3).

As displayed in Table 3, the Wilcoxon signed-rank test showed that the EIM conditions did not elicit a statistically significant change in comparison to baseline [positive EIM: *W*(4) = 2.74, *Z* = -1.83, *p* = 0.07; negative EIM: *W*(4) = 2.74, *Z* = -1.83, *p* = 0.07; and neutral EIM, *W*(4) = 2.73, *Z* = -0.73, *p* = 0.46].

**Table 3:** Raw SCL and HR responses for the four participants congruently induced across the three EIM conditions.

	Baseline	Negative EIM	Positive EIM	Neutral EIM
**Skin conductance level (μS)**		
Participant 1	8.00	9.37	10.04	8.07
Participant 2	8.60	9.13	10.23	8.89
Participant 3	9.00	11.08	9.39	8.61
Participant 4	6.22	9.22	9.27	6.79
**Heart rate (bpm)**		
Participant 1	66.23	64.47	67.54	65.79
Participant 2	82.22	76.52	77.49	82.94
Participant 3	65.68	67.84	64.06	66.34
Participant 4	62.62	68.30	68.49	63.93

As expected, SCL and HR responses during the neutral EIM condition did not differ from baseline, as shown by the above Wilcoxon signed-rank test. The neutral excerpts were poorly rated as having a neutral valence, then we initially contrasted the positive and negative EIM conditions in further within-subject analyses.

### Valence–Arousal Self-Reported, SCL, and HR Responses for Participants Congruently Induced in Positive and Negative EIM Conditions

First, we carried out a power analysis through G^∗^Power 3.1.9.2 (Faul et al., 2007) to verify how many participants would be necessary to perform paired *t*-test analyses (two-tailed). This analysis indicated that with an *n* = 24 there would be sufficient statistical power (β = 0.80) to observe a significant effect (α < 0.05) with a large effect (*dz* = 0.60). For this reason, we included 24 participants in the main analyses, which comprised participants congruently induced on positive and negative EIM conditions (*M*_age_ = 24.12, *SD* = 5.11; 15 women), with self-reported arousal immediately after the induction as the dependent variable and positive and negative EIM as independent variables. The results revealed higher arousal ratings during the positive EIM (*M* = 4.67, *SD* = 1.24) over the negative EIM condition (*M* = 3.96; *SD* = 1.23), *t*(23) = -2.24, *p* = 0.04, *d* = 0.46, 95% CI [-1.36, -0.06]. These results reveal that the EIM procedure used in this study, beyond being effective in inducing happy and sad emotions, which was demonstrated by the valence self-report, it was also accompanied by arousal differences.

Nevertheless, we also conducted power analysis before carrying out repeated-measures within subjects with three (baseline, positive, and negative EIM) and seven measurement times (baseline and 6 min of recovering phase) related to the analyses carried out in the induced emotion across time section, in which the G^∗^Power (Faul et al., 2007) indicated for the first that with *n* = 12 and for the latter with a *n* = 8, there would be sufficient statistical power (β = 0.80) to observe a statistically significant effect (α < 0.05) with an observed medium-to-large effect (*f* = 0.40). In this sense, we included in these analyses the 24 participants mentioned above.

It is important to note that participants underwent only one baseline followed by the counterbalanced positive and negative EIM. For this reason, we applied two independent one-way repeated measures ANOVA including in as independent variables baseline (baseline 3 min mean in silence), positive (EIM 3 min mean), and negative EIM (EIM 3 min mean), and as dependent variables SCL or HR raw responses, in order to confirm whether physiological responses were congruent with self-reported arousal ratings. The results regarding SCL raw responses showed significant differences, *F*(2,46) = 6.62, *p* = 0.003, MSE = 2.16, $\eta_{p}^{2}$ = 0.22, in which negative (Bonferroni; *p* = 0.01) and positive EIM 3-min mean (Bonferroni; *p* = 0.04) showed higher raw responses compared to baseline mean. These findings may be interpreted as a validation of the paradigm itself since it demonstrates the inductive effect of musical stimuli. On the other hand, no significant differences were found for HR raw responses mean, *F*(2,46) = 2.58, *p* = 0.09, MSE = 5.98, $\eta_{p}^{2}$ = 0.10 (Table 4).

**Table 4:** Mean and standard deviation (in parenthesis), and confidence intervals (CI) for participants’ SCL (μS) and HR (bpm) raw responses for baseline, positive, and negative EIM.

	SCL (μS)	95% CI		HR (bpm)	95% CI	
		LB	UB		LB	UB
Baseline	6.87 (3.71)	5.30	8.44	76.06 (10.39)	71.67	80.45
Positive EIM	8.10 (4.32)	6.28	9.93	74.95 (9.13)	71.09	78.81
Negative EIM	8.29 (4.58)	6.35	9.12	74.50 (9.12)	70.64	78.35

*LB, lower bound; UB, upper bound.*

For every participant’s SCL and HR responses, we calculated the *change values* for each 3-min mean during EIM and each one of the 6 min in the recovery phase after the EIM conditions. Change values determine how much each individual changed during EIM in relation to their own baseline. To achieve the change value, we subtracted the baseline total mean from 3-min mean raw responses, of each EIM, e.g., positive EIM:

$$mean_{\left(\text{positive}\;\text{EIM}\right)}\;-\;baseline_{\left(\text{total}\;\text{mean}\right)}.$$

Similar calculations were carried out for the recovery phase, including each one of the 6 min of the recovery phase, after positive and negative EIM. These *change values* served as dependent variables for further analysis.

The effects of EIM conditions after starting musical presentation on SCL and HR *change values* were investigated by two mixed 2 (positive and negative EIM) × 3 (1, 2, 3 min) repeated-measures ANOVAs. A significant effect of minutes was found, *F*(2,46) = 34.52, *p* < 0.001, MSE = 0.32, $\eta_{p}^{2}$ = 0.60, for the analysis including SCL *change values*. Bonferroni pairwise comparisons showed higher SCL responses for the first EIM minute in comparison to the second EIM minute and the third minute (*p*s < 0.001). As well as between the second EIM to the third EIM minute (*p* = 0.004). However, no differences were found between positive and negative EIM, *F*(1,23) = 0.85, *p* = 0.36, MSE = 3.51, $\eta_{p}^{2}$ = 0.03, nor was there a significant interaction, *F*(2,46) = 0.74, *p* = 0.48, MSE = 0.21, $\eta_{p}^{2}$ = 0.03.

Concerning the HR change values, there was no effect of minutes, *F*(2,46) = 0.89, *p* = 0.41, MSE = 6.26, η^2^_p_ = 0.03; condition, *F*(1,23) = 0.54, *p* = 0.46, MSE = 13.63, $\eta_{p}^{2}$ = 0.02; or interaction, *F*(2,46) = 0.97, *p* = 0.38, MSE = 5.32, $\eta_{p}^{2}$ = 0.04 (Table 5).

**Table 5:** Mean and standard deviation (in parenthesis), and confidence intervals (CIs) for participants’ SCL and HR change values on 3 min epoch of the positive and negative EIM.

	SCL (μS)	95% CI		HR (bpm)	95% CI	
		LB	UB		LB	UB
**Positive EIM**		
First minute	1.73 (2.48)	0.68	2.78	-1.60 (6.08)	-4.17	0.97
Second minute	1.11 (2.32)	0.13	2.09	-1.96 (6.08)	-4.53	0.60
Third minute	0.85 (2.23)	-0.09	1.80	-1.51 (5.54)	-3.85	0.83
**Negative EIM**		
First minute	2.04 (2.42)	1.02	3.06	-1.44 (5.59)	-3.81	0.91
Second minute	1.50 (2.25)	0.55	2.45	-2.32 (5.82)	-4.78	0.13
Third minute	1.02 (2.13)	0.12	1.92	-2.66 (6.22)	-5.29	-0.03

*LB, lower bound; UB, upper bound.*

Moreover, to investigate gender differences between HR and SCL change values, we conducted exploratory statistical analyses using a non-parametric test, the Mann–Whitney *U*-Test, and no SCL *change values* differences between genders were observed.

### The Induced Emotion Across Time

We carried out Friedman test analyses for the self-reported arousal rates before the EIM, immediately after the EIM, and after each minute of the recovery state. No differences were observed neither for positive EIM, χ^2^(7) = 8.03, *p* = 0.33, nor negative EIM χ^2^(7) = 8.36, *p* = 0.30.

**FIGURE 3:**
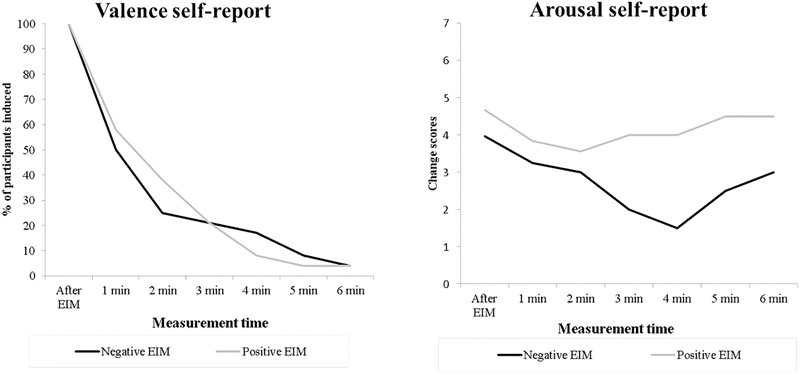
Percentage of participants that remained induced (left) and arousal self-reported rates induced for induced participants (right) across measurement times.

Furthermore, Figure 3 shows the subjective valence (adjectives reported) and arousal responses for each of the seven valence–arousal self-report measurements for both positive and negative EIM conditions of the induced participants. The valence of adjectives reported for both positive and negative EIM changes quickly to neutral ones after the first 2 min. Moreover, the graph with arousal self-reported rates shows the results of participants that remained induced (indicated the same valence adjective).

In order to investigate whether the influence of positive and negative EIM conditions remain during the recovery phase (i.e., at the six specific recovery phase moments – 1 min each), since the assumption of normality was not met across all six recovery phase moments, we performed the analyses with the non-parametric Friedman test, as can be seen below.

The SCL and HR baseline raw responses were compared to the recovery phase raw data after positive and negative EIMs. These analyses were done to confirm whether emotional activations continued throughout the recovery phase after the EIM procedure. The Friedman’s test comparison showed that activations during recovery phase increased with statistical significance for SCL compared to baseline after positive, χ^2^(6) = 31.56, *p* < 0.001, and negative EIM, χ^2^(6) = 29.05, *p* < 0.001 (Table 6).

**Table 6:** Mean and standard deviation (SD) for participants’ SCL and HR raw data in 6 min epoch for the entire duration of the recovery phase for positive and negative EIM.

Recovery phase min	SCL (μS)		HR (bpm)	
	Mean	*SD*	Mean	*SD*
**Recovery phase per minutes after positive EIM**		
First	8.64	4.64	75.93	8.55
Second	8.68	4.59	75.70	8.45
Third	8.71	4.70	74.96	7.99
Forth	8.70	4.87	75.31	8.80
Fifth	8.56	4.83	74.67	8.36
Sixth	8.71	5.04	75.37	8.36
**Recovery phase per minutes after negative EIM**		
First	8.45	4.98	76.87	9.02
Second	8.34	4.48	75.55	9.32
Third	8.39	4.42	76.25	9.83
Forth	8.40	4.42	76.06	9.45
Fifth	8.50	4.55	75.99	10.06
Sixth	8.63	4.53	76.68	9.44

*Post hoc* analyses with Wilcoxon signed-rank tests were conducted with a Bonferroni correction applied, which resulted in a significance level set at *p* < 0.008. Increased SCL raw scores were detected for recovery phase after negative EIM compared to baseline for minute 1, *W*(23) = 248.00, *Z* = 3.61, *p* < 0.001; minute 2, *W*(23) = 249.00, *Z* = 3.54, *p* < 0.001; minute 3, *W*(23) = 247.00, *Z* = 3.89, *p* < 0.001; minute 4, *W*(23) = 250.00, *Z* = 3.82, *p* < 0.001; minute 5, *W*(23) = 250.00, *Z* = 4.36, *p* < 0.001; and minute 6, *W*(23) = 250.00, *Z* = 4.64, *p* < 0.001. Similar results were observed during recovery phase after positive EIM procedure in comparison between baseline and minute 1, *W*(23) = 245.00, *Z* = 4.57, *p* < 0.001; minute 2, *W*(23) = 245.00, *Z* = 4.50, *p* < 0.001; minute 3, *W*(23) = 245.00, *Z* = 4.64, *p* < 0.001; minute 4, *W*(23) = 243.00, *Z* = 3.34, *p* < 0.001; minute 5, *W*(23) = 246.00, *Z* = 3.61, *p* < 0.001; and minute 6, *W*(23) = 244.00, *Z* = 4.57, *p* < 0.001. No significant results were found for HR raw responses in comparison to baseline, after positive χ^2^(6) = 6.17, *p* = 0.40 and negative EIM condition, χ^2^(6) = 3.20, *p* = 0.78.

Furthermore, the subsequent four statistical analyses were conducted separately, in which one included the SCL *change values* during the 3 min of positive EIM and their respective 6 min recovery phase, another with the 3 min of negative EIM and following recovery phase. The same pattern of analyses was carried out for HR *change values*. These statistical analyses did not identify any differences, neither for positive and negative EIM SCL *change values* compared to respective recovery phases (positive EIM, χ^2^(6) = 1.84, *p* = 0.93, and negative EIM, χ^2^(6) = 4.36, *p* = 0.62, nor for change values for HR, positive EIM, χ^2^(6) = 8.61, *p* = 0.19, and negative EIM, χ^2^(6) = 4.01, *p* = 0.67). This means that SCL and HR responses remained stable across all minutes of the recovery phase.

In summary, these results showed that positive and negative MMI conditions increased autonomic sympathetic activity assessed by SCLs, and activations remained observable during the 6 min recovery phase after EIM finishing for positive and negative EIM conditions.

### The Neutral EIM

Exploratory analyses were carried out only for the neutral EIM condition in order to see if the results found in the intra-subject analyses would also be observed in the HR and SCL in an inter-subject analysis. Out of the total sample (*n* = 34), 12 participants reported positive valence adjectives, while 11 reported negative adjectives, and only 11 participants responded to the neutral EIM with neutral adjectives. Non-parametric analyses were carried out to compare arousal ratings, SCL, and HR responses as the dependent variable, between groups, those participants that felt positively, negatively, and neutrally induced by the neutral EIM.

A Kruskal–Wallis *H*-test showed that there was a statistically significant difference in arousal ratings for those participants that felt positive, negative, and neutral, χ^2^(2) = 11.33, *p* = 0.003, with mean rank arousal ratings of 19.25 for positive, 9.86 for negative, and 23.23 for neutral. Moreover, we conducted Mann–Whitney’s *U*-tests to evaluate the difference in arousal ratings. To adjust for multiple comparisons using the Bonferroni correction significance was considered for *p* < 0.016. We found a significant effect for positive and negatively induced groups and to negative and neutrally induced. The mean ranks of positive and negative induced were 15.42 and 8.27, respectively; *U*(23) = 25, *Z* = -2.66, *p* = 0.008, and the mean ranks of negative and neutral were 7.59 and 15.41, respectively; *U*(22) = 17.50, *Z* = -2.90, *p* = 0.004. These results showed that negative arousal ratings were lower than positive and neutral.

Regarding physiological measures, we carried out a Wilcoxon signed-rank test to compare SCL and HR baseline and during musical listening for each group positive, negative, and neutral. Results showed that positively induced (*M* = 6.57, *SD* = 3.61) participants had higher SCL responses compared to baseline (*M* = 5.94, *SD* = 3.26), *W*(12) = 65.00, *Z* = -2.04, *p* = 0.04, and the negatively induced group (*M* = 9.00, *SD* = 4.92) showed increased SCL responses in comparison to baseline (*M* = 7.55, *SD* = 4.53), *W*(11) = 64.00, *Z* = -2.76, *p* = 0.006, while the HR responses for the negatively induced group (*M* = 77.98, *SD* = 11.56) revealed lower HR in relation baseline (*M* = 80.29, *SD* = 11.84), *W*(11) = 0, *Z* = -2.93, *p* = 0.003. No significant differences were observed for participants that reported neutral adjectives to neutral EIM. These results reveal that SCL and HR responses are dependent on how participants interpreted the music, and not simply from musical listening *per se*.

### Recovery Phase

With Friedman test, we compared baseline and every 6 min recovery phase, we found that only the negatively induced group showed higher SCL responses across 6 min, χ^2^(6) = 25.55, *p* < 0.001. A separate analysis for baseline and each minute, with the *p*-value set at *p* < 0.008 (Bonferroni correction), revealed that subjects had higher SCL responses in all recovery phase minutes compared to baseline (*p*s = 0.005). These results indicated that SCL responses were maintained, even if the adjectives reported were no longer congruent with the initially felt valence.

## Discussion

To the best of our knowledge, this is the first study investigating whether positive and negative EIM would evoke happy and sad emotions, respectively, assessed via valence–arousal self-report and autonomic activation measurements, specifically SCL and HR. The originality of the present study lies in the fact that we investigated the persistence of negative and positive emotions during 6 min of recovery phase after each EIM with no emotional instructions. Furthermore, we registered valence and arousal self-report together with SCL and HR physiological measures recognized as sensitive to emotion and emotional changes (Kreibig et al., 2007). The present study is a significant update on the existing literature because no previous study has examined how long the induced mood persists and how the mood would turn back to baseline. Besides, we investigated the effects and the persistence of emotions after a newly designed EIM procedure using songs that were tested regarding its capacity to elicit emotions, especially in Portuguese undergraduate students, instead just selecting musical excerpts from previous studies; otherwise, data generated in other countries with different cultural background could bias our results (Lim, 2016).

Consistent with our first predictions, participant’s self-reported responses revealed that both positive and negative EIM conditions were effective in producing happy and sad emotions, respectively. Besides, higher subjective arousal responses were observed for the positive excerpt when compared to negative ones (Krumhansl, 1997; Etzel et al., 2006; Khalfa et al., 2008; Lundqvist et al., 2009). These findings contradict other studies using emotional stimuli, such as images (Yang et al., 2016). However, it is in congruence with Khalfa et al. (2002) that revealed that musical excerpts evoking fear and happiness are strongly arousing emotions, mainly due to its temporal structure.

Regarding the physiological measurement, the analyses for raw data and change values of HR activations did not show differences between baseline and post-induction, nor between positive and negative EIM conditions, only a tendency for HR deceleration. These results are in line with previous ones (Etzel et al., 2006; Khalfa et al., 2008; Lundqvist et al., 2009), which suggests that listening to musical excerpts was not an attentional demanding or a stressing activity to participants (Taelman et al., 2011). Other potential explanation may be related to the difference between these two autonomic responses. Although the literature has focused on the commonalities among the different physiological modalities (i.e., HR, SCL, cortisol, etc.) treating them as interchangeable measures of the physiological component of an emotion, the different physiological responses are mediated by the interaction between different brain circuits (Norman et al., 2014).

However, our results contrast with Krumhansl (1997) which also used the *Adagio in G minor* from Albinoni composer excerpt with a duration of 3 min. Then, after having cautiously observed the performed induction, we noticed that the author included another sad excerpt from Samuel Barber, the *Adagio for Strings, Op. 11*, which has a different minor tone, which, compared to *Adagio in G minor*, is much slower. We can suggest firstly that divergence in the characteristics of the excerpts might be crucial for HR results since musical features interplay in modulating emotions (Peretz et al., 1998; Sloboda and Juslin, 2001; van der Zwaag et al., 2011). Secondly, cross-cultural comparisons in the literature suggest that emotional responses can be quite differently felt by dissimilar musical cultures (Walker, 1996; Balkwill and Thompson, 1999). Finally, the participants in Krumhansl’s study had formal musical training, which can alter the perception of the musical stimuli, consequently the emotions induced (Kawakami et al., 2013).

The SCL measurement revealed a clear differentiation between baseline (silence) and both positive and negative EIM. These results validate that listeners experienced the emotion reported rather than perceived the emotion in the music (Khalfa et al., 2002; Lundqvist et al., 2009). Nevertheless, our results partially replicate previous studies (Krumhansl, 1997; Khalfa et al., 2002; Khalfa et al., 2008; Lundqvist et al., 2009; van der Zwaag et al., 2011), since the sad excerpt did not decrease SCL responses; instead, it revealed analogous activations of happy excerpt. This negative EIM SCL increase cannot be attributed to gender since both men and women obtained similar activations (Panksepp, 1995).

A critical observation of those studies that detected decreased SCL responses for negative EIM was that they explicitly instructed participants to feel in a specific way prior to the emotional induction. For instance, Lundqvist et al. (2009) in their procedure explained to participants the goals of their study. While Khalfa et al. (2008) and Krumhansl (1997) included in their design a training on how to respond emotionally to music. It is worthwhile to mention that previous investigations showed that telling the participants that they will be asked to rate their mood after an emotional stimulus presentation is sufficient to induce an expected emotional reaction when compared to no guidance before the emotional induction (Westermann et al., 1996).

Another aspect to be taken into account is the multi-faceted emotional experience, which underlies sad music since it is often described by participants as melancholic yet pleasant, and often as unpleasant. In the case of our sample, they felt the negative EIM as sad and unpleasant in congruence with previous studies (Baumgartner et al., 2006; Nater et al., 2006). Otherwise, it would be perceived as calmer and decreases SCL (Mori and Iwanaga, 2017). Future studies should include an investigation regarding pleasantness and chills of sad excerpts to have a broad view of the musical evoked emotions.

Regarding neutral EIM condition, mixed results related to valence were observed, in other words, neutral EIM generated positive and also negative emotions. This confirms the assumption that musical neutrality is rare (Krumhansl, 1997; Peretz et al., 1998) since people listen to music to change how they feel (Shifriss et al., 2015). Interestingly, our exploratory results showed that the subjective-report corroborated SCL since no differences were found just for those participants that responded that they were in a neutral state after hearing the “neutral” song. For this reason, we recommend experimenters to carefully test the participant’s responses and consider for the neutral condition just those that were in a neutral state after listening to the song. Further studies should consider inquiring participants about thoughts generated during EIM to evaluate its valence, and consequently its influence on the evoked emotions.

This study was the first to examine the persistence of an EIM paradigm. According to our valence–arousal self-report, the valenced adjectives changed quickly to neutral ones after the second minute of the recovery phase, which is congruent with Gomez et al. (2009) and Kuijsters et al. (2016), both using videos. However, over time, SCL remained increased at least 4 min more for positive and negative EIM. One possible explanation for this longer-lasting arousal effect could be that, contrary to the physiological measures, the self-report rates are discrete measurements that cannot continually capture all the variations of the emotions felt (Mauss and Robinson, 2009) or participant’s attention to the stimulus decreases.

According to our results, carefully selected positive and negative excerpts can be effective in inducing happy and sad states, respectively. However, positive and negative emotions seem to change quickly to the neutral state after musical exposure. It raises a concern about research undertaking emotion-induced effect on a subsequent cognitive task since hedonic adaptation modulates the emotional state continuously. Based on our findings, upcoming studies should investigate the effects of musical induction on following cognitive functions by applying a task lasting no more than 2 min. Otherwise, performance shall not be related to the emotions evoked by music. In addition, researchers should also report the cognitive task lengths, a factor rarely accounted.

The present study has certain limitations, which constraint the scope of our conclusions. Firstly, findings may be considered preliminary given that in some of the analysis the sample size was small, specifically for the physiological responses since they presented a significant variation among participants. However, we did our best to include in each analysis the number of participants estimated by the power analysis. Secondly, despite our control over musical stimuli and several individual characteristics, it is possible that individual differences linked to musical preferences, and the impact of personality on EIM could influence outcomes as both might be predictors of emotional elicitation through music (Sachs et al., 2015). In this sense, future studies should also address whether the musical preferences, personality, different ages would impact the persistence of emotions evoked by music, and how explicit valence–arousal reports might induce changes by themselves. Finally, the persistence of those emotions could also be assessed during diverse cognitive tasks, for instance with high and low cognitive demands, since it also seems to influence the persistence of evoked emotions (Ribeiro et al., 2018).

## Ethics Statement

The research was approved by Institutional Review Board of the University of Minho (SECSH 009/2014) and was in accordance with the declaration of Helsinki. All Participants gave their written, informed consent prior to inclusion in the study.

## Author Contributions

FR and FS designed the study. FR and PO-S analyzed the data. FR drafted the initial manuscript. All authors contributed to the interpretation of the results, revised the manuscript, and approved its final version.

## Conflict of Interest Statement

The authors declare that the research was conducted in the absence of any commercial or financial relationships that could be construed as a potential conflict of interest.

## Footnotes

1 Portuguese words for positive (feliz, entusiasmado, and eufórico), negative (triste, melancólico, and angustiado), and neutral (neutro, alheio, and indiferente) self-report.
