# Supplementary material for: Emotional Induction Through Music: Measuring Cardiac and Electrodermal Responses of Emotional States and Their Persistence
Source: Front Psychol. 2019 Mar 6;10:451. doi: 10.3389/fpsyg.2019.00451 (PMC6414444; doi:10.3389/fpsyg.2019.00451)
Supplement: Supplementary file 1 [file Data_Sheet_1.docx]

*Supplementary material*

Emotional induction trough music: Measuring cardiac and electrodermal responses of emotional states and their persistence

Fabiana Silva Ribeiro, Flávia Heloísa Santos, Pedro Barbas Albuquerque, Patrícia Oliveira-Silva

*** Correspondence:**Flávia Heloísa Santos

flavia.santos@ucd.ie

**Pilot Study**

A behavioural pilot study was performed to determine the best excerpts to induce positive or negative emotions and neutral state.

*Stimulus Selection*

Positive, negative, and neutral music was carefully selected to ensure their capacity to induce happy, negative emotions, and neutral state, respectively. Initially, we avoided all popular music themes to control possible memory effects. The songs selection criteria for positive valence were erudite songs with major tone and fast tempo, while the negative ones must be composed in minor one and slow tempo. Moreover, the neutral songs were arranged in a minimalist form characterized by simple sonorities, rhythms, and patterns. We have selected the songs for this pilot from previous investigations, considering their induction efficiency. They were three songs with positive valence, four songs with negative valence, and eight neutral songs (Grewe et al., 2010; Västfjäll, 2002). Selected songs are displayed on the following topic.

**Musical excerpts selection**

All songs were edited into 3 minutes excerpts, in which positive and negative excerpts were edited to convey an intense emotional experience.

**Excerpts for negative EIM:**

*Albinoni “Adagio”* excerpt was extracted from the beginning up to the third minute of the original song, linear "fade out" was carried out only at the end of the excerpt (99 ms);

*Edward*Elgar “*Cello* Concerto*in E minor, Op. 85”*, the excerpt was removed from the 0 min 9 s to 3 min 9 s, linear "fade out" was carried out only at the end of the excerpt (2 s 26 ms);

*Mahler “Symphony Nº.5, fourth movement Adagietto”,* the excerpt began from the 5 min 46 s to 8 min 46 s, linear “fade in" and "fade out" were performed respectively, beginning (6 s 51 ms) and end (3 s 33 ms);

*Tchaikovsky “symphony six. Movement4, B minor”*: The excerpts were extracted from the beginning of the piece up to the third minute, no fade out or fade in was applied.

**Excerpts for positive EIM:**

*Bach “Brandenburg concert nº2”:* the excerpt was removed from 0 min to 3 min, linear "fade in" and "fade out" were performed respectively, beginning (1 s 20 ms) to end (6 s 62 ms);

*Mozart “Symphony in C major nº41”*: the excerpt was removed from 0 min to 3 min, linear "fade out" was accomplished only in the excerpt end (1 s 38 ms);

*Vivaldi “Violin concert in E major Op.13 nº12”*, the excerpt was removed from 0 min to 3 min, linear "fade out" was made on in the excerpt final (2 s 85 ms).

**Excerpts for neutral EIM:**

*Holst “The Planets – Saturn, Bringer of Old age”*, the excerpt was removed from the last three minutes of the music and no "fade out" or “fade in” were performed;

*John Adams* “*Common Tones in Simple Time*,” the selected stimulus was extracted from 6 min 42 s to 9 min 42 s of the original song, linear "fade out" was carried out only at the end of the excerpt (99 ms);

*Steven Reich “Variations for Winds, Strings and Keyboards,”* the Excerpt was removed from the first three minutes of the music, linear "fade out" was carried out only at the end of the excerpt (60 ms).

*Dvorak “the Symphony Nº. 9 in E Minor -From the New World,* *Op. 95, B. 178”,* The excerpt began from the 0 min 50 s until the 3 min 50 s, fade out was applied only in the last 60 ms;

*Holst "The Planets: Neptune - the Mystic",* the excerpt was removed from the 0 min 10 s to 3 min 10 s, fade out was applied during the 50 s of the final minute;

*Holst "The Planets: Venus - the Bringer of Peace",* the excerpt was selected from the 0 min 46 s until the 3 min 46 s, “fade in” was applied during 50 ms at the end of the stimulus;

*Verdi "La Traviata Prelude to Act”,* the excerpt was carefully chosen from the first second of the original song up to the third minute, “*Fade out*” was applied at the 40 s until the end of the stimulus.

*Excerpts emotional measurement.*

The effectiveness of each excerpt was determined by the *valence-arousal self-report measure*, in which the arousal scale had 1-6 points. Moreover, the measure and criteria to consider the effectiveness of the musical excerpts were the same described in the main experiment, in which participants had to choose one of the nine adjectives congruently with the specific EIM condition. We considered an effective emotional stimulus those excerpts that were capable of inducing the desired emotion in more than 70 % of the participants, with an arousal rate above 3.00 points.

*Participants*

The participants of the pilot study were 40 undergraduate students with an average age of 21.00 years old (*SD* = 3.46; 36 females). None of these participants had prior musical training or were familiar to the songs. Besides, they did not participate in the main study.

*Procedure*

Participants were allocated into two groups (*n* = 20) and both listened to seven different musical excerpts, each of them was 3 minutes long. One group listened to four sad and three happy excerpts, while the other group listened to seven neutral pieces, presented in a counterbalanced order, and after each excerpt, they were asked to rate their emotional state (valence and arousal) with the *valence-arousal self-report measure*.

*Results*

In the first figure, we display the participant’s percentage valence rates for positive, negative, and neutral songs. According to our data, the three positive excerpts were efficient in inducing more than 70% of the total sample. Also, three negative excerpts effectively induced sad emotions out of four. The results for neutral excerpts revealed that only three out of seven musical excerpts were effective in inducing neutral state (see Figure 1). Also, we present in Table 1, the arousal rates for all positive and negative excerpts, and only for the three neutral excerpts that were more effective in inducing a neutral state.

(Figure 1)

(Table 1)

*Final excerpt selection*

Based on the valence and arousal ratings, we selected the three excerpts most consistently identified by their induction capacity for positive, negative, and neutral conditions.

Regarding positive excerpt, we selected the *Bach “Brandenburg concert nº2*” which presented a high percentage of effectiveness in inducing happiness accompanied by 3.61 arousal rate means. From the four excerpts contemplated to induce sadness, it was possible to notice that the *Tchaikovsky “symphony six, Movement 4, B minor”* presented more negative valence reports. Conversely, this song was not selected to the mean experiment due to the below arousal level (2.95). Instead of this excerpt, we selected the Albinoni “*Adagio*”, the second excerpt was effective in inducing sadness and with 3.38 arousal rate means.

Finally, based on the higher percentage of neutral states and arousal rates, the selected neutral song was the *Steve Reich "Variations for Winds, Strings, and Keyboards."*


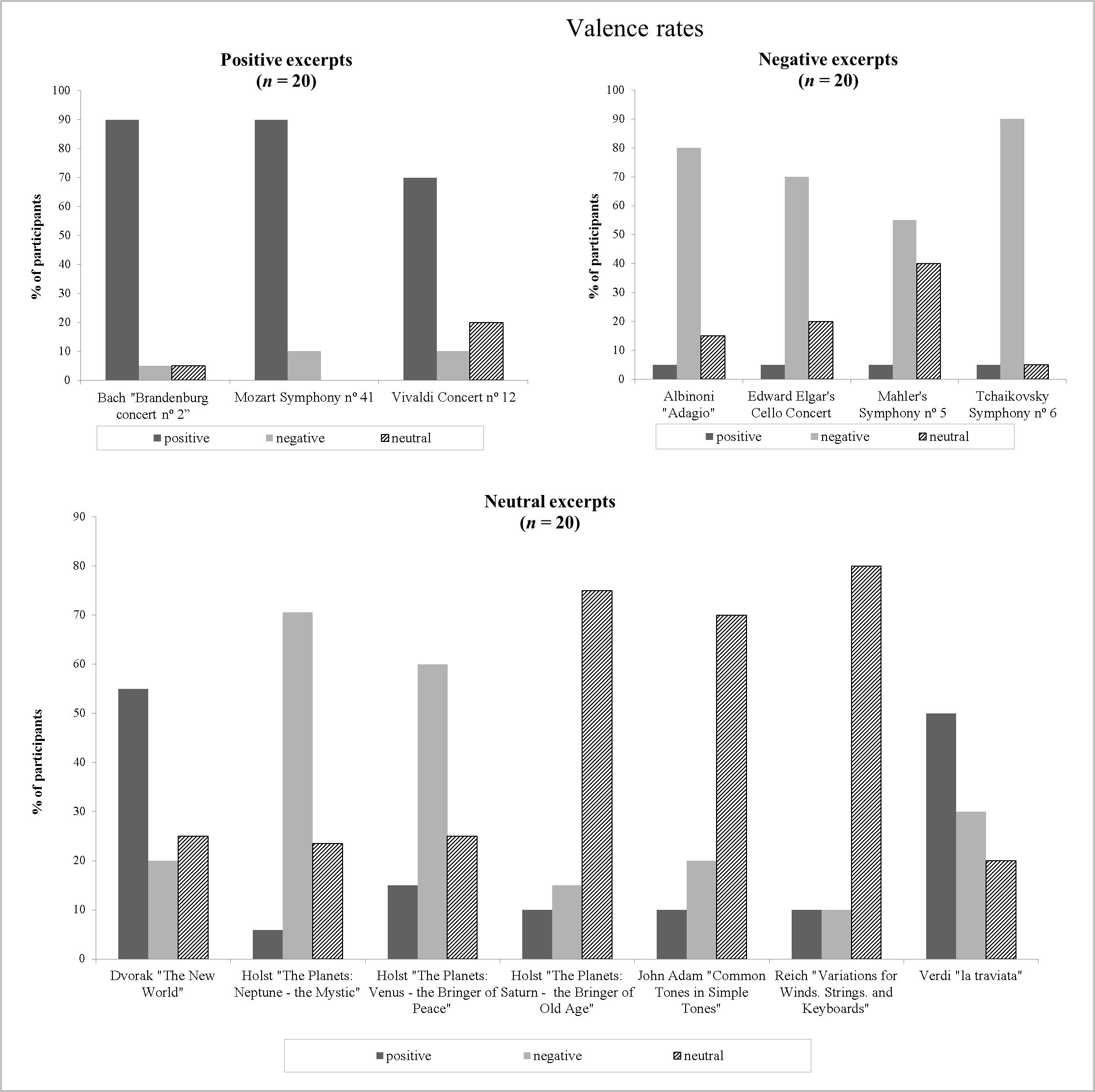


Figure 1. Valence rates for positive, negative, and neutral excerpts.

Table 1

Mean and Standard Deviation (SD) Arousal Rates for All Positive and Negative Excerpts and Three Neutral Excerpts More Effective.

|  | Arousal rates (*n* = 20) | *SD* |
| --- | --- | --- |
| **Positive Excerpts** | |  |
| Bach “Brandenburg concert nº 2 | 3.61 | 0.61 |
| Mozart “Symphony in C major nº 41.” | 3.17 | 0.79 |
| Vivaldi “Violin concert in E major Op.13 nº 12” | 3.62 | 0.77 |
| **Negative Excerpts** | |  |
| Albinoni “Adagio” | 3.38 | 0.89 |
| Edward Elgar “Cello Concerto in E minor, Op. 85” | 3.36 | 1.20 |
| Mahler “Symphony nº 5, fourth movement Adagietto.” | 3.09 | 0.70 |
| Tchaikovsky “Symphony six. Movement 4, B minor.” | 2.95 | 0.70 |
| **Neutral Excerpts** | |  |
| John Adam "Common Tones in Simple Tones" | 4.36 | 0.93 |
| Holst "The Planets: Saturn - the Bringer of Old Age" | 3.71 | 1.12 |
| Steven Reich "Variations for Winds, Strings, and Keyboards" | 4.00 | 1.31 |
